# Performance validation of Abbott Alinity i chemiluminescence analyzer for five thyroid function tests

**DOI:** 10.1016/j.plabm.2025.e00487

**Published:** 2025-06-25

**Authors:** Hongyu Zhang, Baixiu Wu, Liuhua Ke, Zheng Peng

**Affiliations:** a Department of Clinical Laboratory, The Third Affiliated Hospital of Guangxi University of Chinese Medicine, Liuzhou Traditional Chinese Medical Hospital, The Third Clinical Faculty of Guangxi University of Chinese Medicine, Liuzhou, Guangxi, China; b Department of Nuclear Medicine Department, The Fourth Affiliated Hospital of Guangxi Medical University, Liuzhou Worker's Hospital, Liuzhou, Guangxi, China

**Keywords:** FT3, FT4, TT3, TT4, TSH, Alinity, Performance validation

## Abstract

**Objective:**

To verify and evaluate the performance of the Abbott Alinity i chemiluminescence analyzer for five thyroid function tests.

**Method:**

Referring to the relevant documents of the Clinical and Laboratory Standards Institute (CLSI) and related literature, the precision, accuracy, linear range, reference interval, and sample carryover effect of Abbott Alinity i immunoassay system for measuring FT3, FT4, T3, T4, and TSH were verified and analyzed.

**Results:**

Precision: Repeatability ranged from 1.23 % to 6.11 %, and intermediate precision ranged from 1.84 % to 7.33 %, both meeting the quality targets (≤6.25 % and ≤8.33 %, respectively). Accuracy: The deviation between the mean value and the target value was less than 12.5 %, indicating good agreement. Linearity: For T3, T4, and TSH, the 95 % confidence intervals of the deviation from linearity (difference between measured and expected value) were entirely within the allowable deviation limits (ADL). For FT3 and FT4, manufacturer guidelines preclude dilution; thus, linearity verification was omitted. Reference interval: All test values of 20 healthy individuals were within the reference intervals provided by the manufacturer. Sample carryover effect: The sample carryover effect ranged from −0.4 % to 0.17 %, which met the requirement of less than 1 %.

**Conclusion:**

The Abbott Alinity i analyzer demonstrated acceptable performance in precision, accuracy, linearity (for T3, T4, and TSH), reference interval, and carry-over effect for the five thyroid function tests, meeting manufacturer specifications.

## 1 Introduction

The thyroid gland is an important endocrine organ in the human body, and its primary function is to regulate the body's metabolism. Thyroid dysfunction is the most common endocrine disorder, and its causes are complex. If left untreated, it may cause serious adverse health effects on multiple organ systems [1]. Thyroid hormones (TH) are involved in a variety of physiological processes, including cell growth, embryonic development, differentiation, metabolism, and proliferation [2]. TSH, free triiodothyronine (FT3), free thyroxine (FT4), total triiodothyronine (TT3), and total thyroxine (TT4), are commonly used laboratory indicators to determine the functional status of the thyroid gland. These five indicators are of great value in determining the type of thyroid disease, monitoring the efficacy of treatment, and evaluating patients' conditions, which directly affect the clinician's diagnosis and medication prescriptions. The literature has suggested that TSH acts as a growth factor for thyroid follicular cells (including those that are neoplastic); it can potentially affect the onset and/or progression of follicular cell-derived thyroid cancer [3]. A retrospective study by Krashin et al. noted that abnormal thyroid function is associated with increased cancer mortality, meaning that it has the potential to be a prognostic marker for cancer [4]. Furthermore, type 2 diabetes risk is elevated in people with hypothyroidism and lower FT4 levels in the reference range [5]. Therefore, as an important indicator for clinical diagnoses and the identification of diseases, TH-related test results and their accuracy are important in the development and prognosis of diseases.

Accurate and reliable thyroid function test results are crucial for the diagnosis, treatment, and monitoring of thyroid diseases. The environmental conditions in the laboratory (temperature, humidity, electromagnetic fields, noise, etc.) may not be completely consistent with the environmental conditions verified by the instrument manufacturer, which may cause the performance of the reagents to deviate from that stated in the instructions. To ensure the accuracy and reliability of the results, a performance validation program was developed in accordance with the requirements of ISO 15189:2022 “Medical laboratories—Requirements for quality and competence” and with reference to the standards set by the Clinical Laboratory Standards Institute (CLSI) [[6], [7], [8], [9], [10]]. The performance of the above five items was verified and evaluated in terms of precision, accuracy, linearity range, reference interval, and sample carry-over effect.

## 2 Materials and methods

### 2.1 Instruments and reagents

The instruments used in this study were the Abbott Alinity i fully automated chemiluminescence analyzer. Supporting reagents and calibrators were used for he Abbott Alinity i fully automated chemiluminescence analyzer. IA Plus ImmunoQC Levels 2 and 3 (lot number: 036604220) were required for the experiments. In addition, five National Endocrine Inter-Room Quality Assessment (NIRQA) specimens were required (lot numbers 202221, 202222, 202223, 202224, and 202225). All equipment was operated strictly according to the standardized operating procedures provided by the manufacturer.

### 2.2 Performance verification

After professional training, the laboratory personnel were able to calibrate the instruments, engage in quality control, and maintain the instruments according to the requirements of the operation manual. The laboratory passed the ISO15189 medical laboratory accreditation. According to the relevant documents, it is necessary to verify the performance of the testing items of the new system. This is mainly carried out in the ways described below.

#### 2.2.1 Precision

Precision was evaluated according to CLSI EP5-A2 guidelines [7]. External quality control (QC) samples were categorized into three levels: low (level 1), medium (level 2), and high (level 3). Aliquots of each QC level were stored at −20 °C.

**Repeatability:** Each QC level (low, medium, and high) was analyzed in 20 replicates within a single analytical run (same operator, reagent lot, and calibration). The coefficient of variation (CV%) was calculated for each level.

**Intermediate precision:** QC samples were tested once daily over 20 consecutive days, with at least one reagent lot change during this period. Calibration was performed after each lot change. The total CV% was calculated across all runs to assess between-run variability.

Data analysis was performed using Analyze-It software version 2.22 (Analyze-it Software Ltd, Leeds, UK). The TEa for the thyroid function tests in this study was set at 25 % by the proficiency testing plan provider (the National Center for Clinical Laboratories). Therefore, the quality objectives were set as follows: repeatability ≤6.25 % (1/4 × 25 %) and intermediate precision ≤8.33 % (1/3 × 25 %).

#### 2.2.2 Accuracy

Accuracy was performed to evaluate the agreement between the Abbott Alinity i system and target values assigned by the National Health Commission Clinical Laboratory Center. Five quality control (QC) specimens from the second round of the 2022 National Endocrine Inter-Laboratory Quality Assessment Program were analyzed. Each specimen was tested in triplicate, and the mean measured value was compared to the assigned target value. The allowable bias was set at ≤12.5 % (1/2 × 25 %) based on CLSI EP15-A2 recommendations. The National Health Commission Clinical Laboratory Center assigns target values to QC samples through a standardized and traceable process: Multi-Laboratory Consensus. For analytes lacking internationally recognized reference methods, target values are derived as the robust mean of results obtained from ≥20 accredited laboratories participating in the program and using harmonized protocols and various validated assay systems. Outliers are excluded using robust statistical methods (e.g., Tukey's test). Therefore, the target value represents a peer-group consensus value, not a value specific to any single instrument like the Alinity i.

#### 2.2.3 Linearity verification

The linear interval is verified according to CLSI EP06-ED2. The samples at the upper and lower limits of the linear interval are collected as high-value (H) and low-value (L) samples, respectively. H and L samples are mixed in proportion. A total of seven concentration gradients are prepared: H, 5/6H + 1/6L, 4/6H + 2/6L, 3/6H + 3/6L, 2/6H + 4/6L, 1/6H + 5/6L, L.

Each sample is measured twice, and the mean and standard deviation are calculated. The allowable deviation from linearity (ADL) percentage is set to 1/2 TEa, which is 12.5 % (1/2 × 25 %). The ADL is calculated based on the expected value of each sample, using the formula: ADL = ± (expected value × 12.5 %). When the 95 % confidence interval of the linear deviation of all samples intersects with the ADL, the linearity of the entire interval is acceptable, meaning the linear interval is verified.

#### 2.2.4 Reference interval validation

According to the CLSI C28-A3 guidelines [10], a total of 20 healthy individuals were recruited from the Preventive Health Center of Liuzhou Traditional Chinese Medical Hospital for reference range validation. Participants were aged 25–55 years (mean ± SD: 38.2 ± 8.7 years) and represented a mix of 12 females (60 %) and 8 males (40 %).

They were asymptomatic outpatients undergoing voluntary annual health screenings and were not referred from specialty clinics (e.g., endocrinology). To ensure the accuracy of the reference range validation, participants were carefully screened for the following criteria:Health Status: No self-reported history of thyroid disease, autoimmune disorders, or endocrine/metabolic diseases (e.g., diabetes); normal results for routine laboratory tests (complete blood count, liver/kidney function, fasting glucose) and thyroid ultrasound. Medication History: No use of thyroid-related medications (e.g., levothyroxine, antithyroid drugs) or hormonal therapies within the past 6 months.

#### 2.2.5 Validation of the sample carry-over effect

Referring to the CLSI EP10-A [11] document, one sample each for H and L concentrations was used for testing. The high-value specimen was tested three times continuously (H1, H2, and H3), and the low-value specimen was tested three times immediately (L1, L2, and L3). The sample carry-over effect was calculated according to the following formula: sample carry-over effect (%) = (L1 – L3)/(H3 – L3) × 100 %. The sample carry-over effect judgment standard is <1 %.

### 2.3 Statistical processing

The Statistical Package for the Social Sciences (SPSS) version 25.0 was used for statistical analysis. Measurement data were expressed as the mean ± standard deviation, and the *t*-test was used for inter-group comparisons; count data were expressed as the rate, and the χ^2^ test was used for inter-group comparisons. In the linear regression analysis, the correlation coefficient r ≥ 0.975 was taken as a good correlation. The difference was considered statistically significant at *P* < 0.05.

## 3 Results

### 3.1 Precision results

As shown in Table 1, the precision of the Abbott Alinity i system was evaluated in terms of repeatability and intermediate precision. As shown in Table 1, repeatability CV% ranged from 1.23 % to 6.11 %, and intermediate precision CV% ranged from 1.84 % to 7.33 %, meeting the predefined precision goals (≤6.25 % for repeatability and ≤8.33 % for intermediate precision).

**Table 1:** Precision results of five items of thyroid function (%) (TEa∗ = 25 %) (n = 20).

Item	Level	Mean	Repeatability		Intermediate Precision	
			CV (%)	95 % CI	CV (%)	95 % CI
FT3 (pmol/L)	1	2.33	4.45	4.2–6.4	4.68	5.3–7.9
	2	4.63	1.45	2.1–3.8	2.21	3.7–4.9
	3	9.97	2.67	1.4–2.7	3.23	2.8–4.4
FT4(pmol/L)	1	8.18	5.47	4.1–5.9	7.33	5.7–7.3
	2	14.67	2.27	3.5–5.1	2.15	3.1–4.9
	3	33.54	2.16	2.2–4.2	4.57	2.8–4.7
TT3 (nmol/L)	1	0.41	6.11	4.3–6.6	5.23	3.9–6.7
	2	1.40	1.92	3.2–5.1	2.19	3.7–5.5
	3	3.82	1.92	1.3–3.8	3.22	2.4–4.7
TT4 (nmol/L)	1	44.2	4.33	3.4–5.7	4.68	4.1–5.7
	2	98.3	1.23	2.6–4.4	2.68	3.1–5.0
	3	182.2	1.87	1.8–3.1	2.86	2.1–3.9
TSH(IU/L)	1	0.29	5.14	4.2–6.4	5.77	4.4–7.1
	2	3.97	1.93	3.5–4.9	1.84	3.9–5.4
	3	23.25	1.68	2.1–3.7	3.07	2.9–4.7

TEa∗: total allowable error.

The TEa for this study was set by the proficiency testing plan provider (the National Center for Clinical Laboratories).

### 3.2 Accuracy results

The accuracy of five thyroid function tests … was evaluated using quality control (QC) samples from the 2022 National Endocrine Inter-Laboratory Quality Assessment Program (National Health Commission Clinical Laboratory Center). The QC materials were lyophilized human serum pools. Target values were assigned by the program provider using a multi-laboratory consensus process, defined as the robust mean of results from ≥20 accredited laboratories using various validated assay systems. The mean bias (%) was calculated from triplicate measurements (n = 3 per sample) using the formula: Bias (%) = (Mean Measured Value - Target Value)/Target Value × 100 %.A bias ≤12.5 % (1/2 TEa) was deemed acceptable per CLSI EP15-A3 guidelines. The validation was successful, and the results are shown in Table 2.

**Table 2:** Method comparison results for thyroid function tests using National Health Commission quality control samples.

Sample ID	Item bias					Judgment criteria	Conclusion
	FT3	FT4	TT3	TT4	TSH		
202221	−0.97	3.26	4.53	−4.77	−4.95	≤12.5	Acceptable
202222	−2.67	−3.38	−6.90	3.09	9.76	≤12.5	Acceptable
202223	4.06	3.10	1.50	−1.14	−2.17	≤12.5	Acceptable
202224	3.68	4.01	4.27	8.03	0.39	≤12.5	Acceptable
202225	−0.49	−2.24	5.72	−3.86	1.85	≤12.5	Acceptable

### 3.3 Linearity verification

The 95 % confidence intervals (CIs) for the linear deviation of all samples fall within the ADL range. According to the criteria for evaluation (the linearity of the entire interval is considered acceptable when the 95 % CIs of the linear deviation for all samples intersect with the ADL), the linearity validation for T3, T4, and TSH has been successfully passed. For FT3 and FT4, as per the reagent instructions, the manufacturer does not recommend diluting samples with a diluent; therefore, linearity validation for these two tests was not conducted. The results are shown in Table 3.

**Table 3:** Linearity validation results for T3, T4, and TSH.

Items	Mixing ratio	Expected value	Linearity bias	95 %CI		ADL(±%)
				Lower	Upper	
T3	H	9.03	0.01	−0.37	0.38	±1.13
	5/6H + 1/6L	7.65	−0.24	−0.55	0.07	±0.96
	4/6H + 2/6L	6.26	−0.08	−0.34	0.17	±0.78
	3/6H + 3/6L	4.88	−0.17	−0.37	0.03	±0.61
	2/6H + 4/6L	3.49	−0.01	−0.16	0.13	±0.44
	1/6H + 5/6L	2.11	0.00	−0.08	0.09	±0.26
	L	0.72	0.08	0.05	0.11	±0.09
T4	H	306.65	−5.70	−18.32	6.92	±38.33
	5/6H + 1/6L	262.03	2.32	−8.76	13.40	±32.75
	4/6H + 2/6L	217.41	−9.71	−18.41	−1.00	±27.18
	3/6H + 3/6L	172.79	−4.84	−11.88	2.21	±21.60
	2/6H + 4/6L	128.16	−2.16	−7.45	3.12	±16.02
	1/6H + 5/6L	83.54	−3.34	−6.70	0.02	±10.44
	L	38.92	0.33	−1.32	1.98	±4.87
TSH	H	98.93	−1.88	−5.94	2.19	±12.37
	5/6H + 1/6L	82.46	−0.46	−3.90	2.98	±10.31
	4/6H + 2/6L	66.00	−1.29	−4.00	1.43	±8.25
	3/6H + 3/6L	49.53	−0.73	−2.78	1.31	±6.19
	2/6H + 4/6L	33.06	−0.16	−1.53	1.22	±4.13
	1/6H + 5/6L	16.59	−0.17	−0.86	0.52	±2.07
	L	0.13	−0.01	−0.01	0.00	±0.02

95%CI: 95 % confidence interval; ADL:allowable deviation from linearity.

### 3.4 Reference interval validation

In accordance with the requirements of the NCCLS C28-A2 document, specimens from 20 healthy individuals who passed the physical examination were selected for testing. The results showed that the test values of the five items reflecting thyroid function were all distributed within the reference interval provided by the manufacturer. This indicates that the reference interval can be used in the laboratory. The results are shown in Table 4.

**Table 4:** Validation results for reference intervals.

Items	Reference interval	n	R (%)
FT3 (pmol/L)	2.89–4.88	20	100
FT4 (pmol/L)	9.01–19.05	20	100
TT3 (nmol/L)	0.54–2.96	20	100
TT4 (nmol/L)	62.68–150.84	20	100
TSH (μIU/mL)	0.35–4.94	20	100

Note: n is the number of people whose test values are within the reference interval; R = (number of healthy individuals with detection values within the reference interval)/(total number of individuals) x 100 %.

### 3.5 Sample carry-over effect

The sample carry-over effect ranged from −0.4 % to 0.17 %, and the results (Table 5) met the requirements.

**Table 5:** Validation results for the sample carry-over effect of five items of thyroid hormones.

Items	High-value specimens			Low-value specimens			Sample carry-over effect	Requirement
	H1	H2	H3	L1	L2	L3		
FT3 (pmol/L)	13.55	13.68	13.61	2.31	2.37	2.35	−0.36 %	<1 %
FT4 (pmol/L)	46.49	47.27	46.94	5.33	5.38	5.26	0.17 %	<1 %
TT3 (nmol/L)	8.58	8.66	8.81	0.41	0.43	0.40	0.12 %	<1 %
TT4 (nmol/L)	258.22	269.31	260.55	29.62	31.33	30.53	−0.40 %	<1 %
TSH (μIU/mL)	39.25	39.02	39.87	0.21	0.23	0.24	−0.08 %	<1 %

## 4 Discussion

The Abbott Alinity i fully automated chemiluminescent immunoassay analyzer utilizes the principle of chemiluminescent microparticle immunoassay (CMIA), combining the chemiluminescent system with immune reactions to detect antigens, antibodies, and analytes. Compared to traditional methods, the analyzer offers many advantages, such as excellent sensitivity, simple operation, fast detection speed, and no radiation pollution. Good instrument performance and testing methods are the cornerstone of ensuring the accuracy of test results. Therefore, based on the requirements of ISO 15189 and the CLSI, this study developed a performance validation plan for the five thyroid function tests and compared it with other analysis systems.

Precision is the degree of consistency between the results of repeated testing of the same specimen, which is typically represented by the coefficient of variation. Precision is one of the most important performance analysis indicators in a testing procedure. The study results demonstrated that for the five thyroid function items, the Alinity i chemiluminescence immunoassay analyzer achieved repeatability below 1/4 TEa (CLIA ‘88) and intermediate precision below 1/3 TEa (CLIA ‘88). This confirms the instrument's strong analytical performance and compliance with established quality standards. There is also a report in the literature that the sigma value of the Alinity i system is greater than 5, confirming that its precision has reached the world's leading level [12,13].

Accuracy is the degree of agreement between the mean value of a test result and a reference value (approximate true value), and it is often expressed as bias. The lower the bias, the higher the agreement. The laboratory used original Abbott reagents and calibrators, so the system had good traceability. The results of the agreement validation showed that the bias between the mean values of FT3, FT4, TT3, TT4, and TSH and the target values was less than 1/2TEa, indicating that the instrument's measurements were highly reliable and could meet the clinical requirements. Nam et al. also pointed out that the precision of Alinity i is excellent in detecting infectious disease programs, which is consistent with the findings of this study [14].

The analytical measurement range was verified according to the CLSI EP06-ED2 document. The analytical measurement range verification confirmed good linearity for T3, T4, and TSH within their respective ranges. For FT3 and FT4, linearity was not assessed due to manufacturer restrictions on dilution. Linearity for FT3 and FT4 was not evaluated as per manufacturer guidelines, which may limit the interpretation of results for samples requiring dilution. Future studies could explore alternative methods to assess linearity for these analytes.

Reference intervals provide important evidence for assessing the clinical results of the analysis. The results for different items may be affected by factors such as age and gender, so it is necessary to validate the reference intervals provided by the manufacturer. The results for different items may be affected by factors such as age and gender, so it is necessary to validate the reference intervals provided by the manufacturer. The laboratory selected 20 healthy individuals for the five thyroid function tests, and 100 % of the results were within the reference intervals. The results of the reference interval validation were satisfactory, meaning that the reference intervals provided by the manufacturer can be used directly. Although reference interval validation was passed, TH are significantly affected by gender, age, iodine intake, sample size, region, assay method, and manufacturer [15]. Therefore, it is necessary for laboratories to determine appropriate reference intervals for TH based on local conditions. Reference values and cutoff values are important tools in clinical laboratory testing for evaluating results and diagnosing diseases. Reference values represent the numerical distribution range of a healthy population and are used to determine whether an individual's test result is within the normal range; while cutoff values serve as the dividing point between normal and abnormal, used to identify disease states. In clinical practice, it is important to note that reference value ranges are not absolute and are influenced by various factors; cutoff values also have an error range; individual differences need to be considered comprehensively; and some indicators require dynamic observation. Understanding and applying these concepts can help clinicians and laboratory personnel more accurately interpret test results and provide more reliable diagnostic and treatment services for patients.

The sample carry-over effect is the interaction between samples of different concentrations in the detection process. A case of interference with a high-value human chorionic gonadotropin (HCG) specimen from a normal specimen occurred in our laboratory, mainly due to incomplete cleaning of the spiking needle by the flushing component. In this study, the sample carry-over effect was verified to be <1 %, which meets the requirements. We acknowledge that in our current study, we did not evaluate other possible sources of contamination, such as carryover from reagents or the instrument itself. To overcome this limitation in our research, future studies need to delve into these potential additional sources of contamination.

In this study, we evaluated the performance of thyroid function tests (TFT) on the Alinity i system and found that it demonstrated good performance in terms of imprecision, linearity, reference intervals, and sample carryover, which is consistent with the findings of Sten Westgard et al. on the Alinity ci system [16]. Their study showed that 37 clinical chemistry assays, 13 immunoassays, and 3 ICT methods on that system achieved Sigma metrics no less than three Sigma, with most assays exceeding five Sigma, indicating that laboratories can expect excellent or world-class performance. Similarly, research by Sunyoung Ahn supports our findings, noting that TFT on the Alinity i system exhibited acceptable performance in precision, linearity, comparison, functional sensitivity, and carryover [13]. However, it is important to note that, as highlighted by Wigh et al. [17], TFT results obtained from different immunoassays are not interchangeable. These discrepancies significantly impact clinical decision-making and emphasize the importance of clinical laboratory information when using different assays for diagnosing and monitoring patients with thyroid disorders. In summary, TFT on the Alinity i system meets the manufacturer's specifications and fulfills the requirements for clinical testing.

Although our study demonstrates that deviations in FT4 measurements using quality control (QC) materials remain acceptable (within 1/2 TEa), it is important to note that significant variability persists across immunoassay systems for thyroid function indicators. These inconsistencies directly undermine the accuracy of clinical decision-making, highlighting unresolved technical limitations rooted in method-specific analytical interference, reagent nonspecificity, and autoantibody interference [18,19]. To address this, the International Federation of Clinical Chemistry (IFCC) Thyroid Function Testing Standardization Committee, in collaboration with the U.S. CDC and other institutions, has established a conventional reference measurement procedure (cRMP) for free thyroxine (FT4) based on equilibrium dialysis principles and validated clinical sample correlations, achieving preliminary standardization and comparability of FT4 test results [20]. While our validation confirms the Abbott Alinity i meets manufacturer specifications for FT4 accuracy within the evaluated range, recent evidence reveals significant platform-dependent differences at elevated concentrations. Studies comparing Abbott Alinity with Roche Cobas, Siemens ADVIA Centaur, and equilibrium dialysis LC-MS/MS demonstrate that Alinity yields markedly lower FT4 values in hyperthyroid samples [21]. This non-linear bias could lead to underestimation of disease severity if clinicians rely solely on absolute FT4 values. Consequently, guideline-recommended antithyroid drug dosing adjustments—based on FT4 multiples of the upper reference limit—require method-specific interpretation. Laboratories should clearly flag FT4 results with the assay platform in medical records (e.g., ‘FT4 [Abbott Alinity]’) to prevent clinical misinterpretation. However, standardization efforts face critical challenges: current progress remains largely focused on FT4, while inter-system variability in measuring FT3, TSH, and other parameters persists, and routine quality assessments continue to reveal significant inter-laboratory discrepancies [22]. The lack of standardization has negatively impacted cross-platform result consistency, necessitating clinical laboratories to explicitly report methodological details and adopt unified interpretive criteria. Moving forward, expanding standardization to encompass all thyroid-related biomarkers through technological innovations (e.g., validation of mass spectrometry) and global collaboration will be essential to enhance result comparability and establish a unified, reliable diagnostic foundation for evidence-based medicine.

While the study provides valuable insights into the performance of the Abbott Alinity i chemiluminescence immunoassay analyzer for thyroid function tests, it also acknowledges several limitations:(1)Limited evaluation of contamination sources: The study only assessed the sample carry-over effect as a potential source of contamination. It did not investigate other potential sources, such as carryover from reagents or the instrument itself. This leaves open the possibility of other contamination mechanisms impacting the accuracy of results.(2)Limited sample size for reference interval validation: The study used only 20 healthy individuals to validate the reference intervals provided by the manufacturer. While this met the minimum requirement of 90 % of results falling within the reference interval, a larger sample size would provide more robust and representative data.(3)Lack of comparison with other methods: While a direct comparison with other assay systems was not conducted in this study due to the decommissioning of the Beckman DXI800 analyzer at our laboratory, previous literature has demonstrated the good agreement of the Alinity i system with other chemiluminescence platforms in infectious disease and endocrine testing [23,24]. Future research will prioritize cross-method comparisons to further validate these findings. Suggestions for future research: (1)Investigate other potential sources of contamination: Future studies should explore potential contamination from reagents and the instrument itself to ensure comprehensive evaluation of the impact on accuracy.(2)Increase sample size for reference interval validation: A larger sample size would provide more robust and representative data for reference interval determination.

(3)Control for external factors: Future studies should consider and control for potential confounding factors to ensure accurate interpretation of results. Despite its many shortcomings, this study is the first all-encompassing assessment of the performance of Abbott Alinity i.

In conclusion, the Abbott Alinity i analyzer demonstrated acceptable performance in precision, accuracy, linearity (for T3, T4, and TSH), reference interval validation, and carry-over effect for the five thyroid function tests. These results meet the manufacturer's specifications and comply with the requirements of CLIA ‘88 and ISO15189 laboratory accreditation.

## CRediT authorship contribution statement

**Hongyu Zhang:** Resources, Methodology, Investigation, Funding acquisition, Formal analysis, Data curation, Conceptualization. **Baixiu Wu:** Resources, Project administration, Methodology, Investigation, Funding acquisition, Formal analysis. **Liuhua Ke:** Visualization, Validation, Supervision, Software, Resources, Project administration. **Zheng Peng:** Writing – review & editing, Writing – original draft, Visualization, Validation, Software, Resources.

## Informed consent

Informed consent was obtained from all participants.

## Ethical approval

The study was approved by the Hospital Ethics Committee of Liuzhou Traditional Chinese Medicine Hospital.

## Research funding

This work was supported by the Guangxi Zhuang Autonomous Region Health and Wellness Commission Self-financed Research Project Fund (Z-B20231492).

## Declaration of competing interest

We, the authors of the manuscript titled “Performance Validation of Abbott Alinity i Chemiluminescence Analyzer for Five Thyroid Function Tests,” declare that we have no conflicts of interest to disclose. This research was conducted independently and was not influenced by any commercial, financial, or personal relationships that could be perceived as a potential bias. The funding for this study was provided by the Guangxi Zhuang Autonomous Region Health and Wellness Commission Self-financed Research Project Fund (Z-B20231492), and it did not involve any industry sponsors. We have adhered to the ethical guidelines and standards of our institution and the relevant professional organizations throughout the research and writing process.

## Data Availability

Data will be made available on request.
